# Supplementary material for: Identification of photoperiod-induced specific miRNAs in the adrenal glands of Sunite sheep (Ovis aries)
Source: Front Vet Sci. 2022 Jul 22;9:888207. doi: 10.3389/fvets.2022.888207 (PMC9354845; doi:10.3389/fvets.2022.888207)
Supplement: Supplementary file 1 [file Data_Sheet_1.ZIP › Supplementary file/miRDeep2 file.zip!/miRDeep2 file/Novel_Structure/SPLP42A1/Novel_666.pdf]

Provisional ID	:	NC_019458.2_1144
Score total	:	1.4
Score for star read(s)	:	-1.3
Score for read counts	:	0
Score for mfe	:	1.1
Score for randfold	:	1.6
Score for cons. seed	:	
Total read count	:	3570
Mature read count	:	3570
Loop read count	:	0
Star read count	:	0

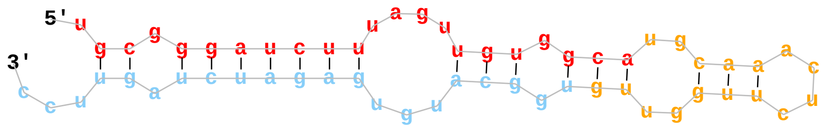

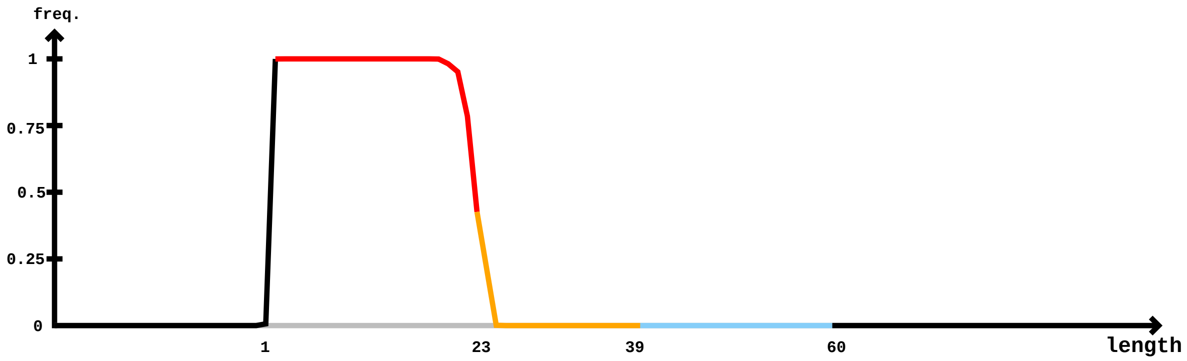

Mature **Star**

5'- ucgucagucuuuguuuuggguugcgggaucuuuaguuguggcaugcaaacucuugguuguggcaugugagaucuaguucccaacaagggauggaugcuuacuccuuggaaga -3'	exp		
.((((.((((((...(((((..((.(((((((....(((.(((..(((....)))..))).)))...))))))).))..)))))..)))))).))))((..(.....)..)).	reads	mm	sample
....................Cugcgggaucuuuaguugu..	1	1	seq
....................Augcgggaucuuuaguuguggca..	21	1	seq
....................uugcgggaucuuuaguuguggca..	1	0	seq
.....................ugcgggaucuuuaguug...	2	0	seq
.....................ugcgggaucuuuaguugA..	7	1	seq
.....................ugcgggaucuuuaguugu..	56	0	seq
.....................ugcgggaucuuuaguugug...	76	0	seq
.....................ugcAggaucuuuaguugug...	2	1	seq
.....................uAcgggaucuuuaguugug...	1	1	seq
.....................ugcgggaucuuuaguuguA...	28	1	seq
.....................ugcgggaucuuuaguugugU..	24	1	seq
.....................ugcgggaucuuuaguugugg..	422	0	seq
.....................ugcgggaucuuuaguugCgg..	1	1	seq
.....................uAcgggaucuuuaguugugg..	3	1	seq
.....................ugcAggaucuuuaguugugg..	8	1	seq
.....................ugcgggaucuuuaguugugA..	137	1	seq
.....................ugcgggaucuuuaguuguggU...	390	1	seq
.....................ugcAggaucuuuaguuguggc...	14	1	seq
.....................ugcgggaucuuuaguuguggc...	869	0	seq
.....................uAcgggaucuuuaguuguggc...	6	1	seq
.....................ugGgggaucuuuaguuguggca..	1	1	seq
.....................ugcgggaucuuuaguugugAca..	29	1	seq
.....................ugcgggaucuuuaguuguggUa..	14	1	seq
.....................ugcgggaucuuuaguuguggca..	1334	0	seq
.....................uAcgggaucuuuaguuguggca..	7	1	seq
.....................ugcgggaucuuuaguugCggca..	5	1	seq
.....................ugcgggaucuuuagCuguggca..	2	1	seq
.....................ugcgAgaucuuuaguuguggca..	5	1	seq
.....................ugcAggaucuuuaguuguggca..	12	1	seq
.....................ugcgggaucuuuaguuguggcC..	62	1	seq
.....................ugcgggaucuuuaguugugUca..	1	1	seq
.....................ugcgAgaucuuuaguuguggcau...	1	1	seq
.....................ugcAggaucuuuaguuguggcau...	3	1	seq
.....................ugcgggaucuuuaguuguggcaC...	10	1	seq

```
                     Mature                                 Star
ucgucagucuuuguuuuggguugcgggaucuuuaguuguggcaugcaaacucuugguuguggcaugugagaucuaguucccaacaagggauggaugcuuacuccuuggaaga
.....................ugcgggaucuuuaguuguggcCu.....................................................................    10    1    seq
.....................ugcgggaucuuuaguuguggcauA....................................................................    3     1    seq
.....................ugcgggaucuuuaguuguggcaug....................................................................    1     0    seq
......................gcgggaucuuuaguuguggc.......................................................................    1     0    seq
```
